# Mediating mechanism of posttraumatic growth as buffers of burnout and PTSD among nurses during the COVID-19 pandemic

**DOI:** 10.3389/fpubh.2024.1406514

**Published:** 2024-07-05

**Authors:** Jae-Chang Sim, Sun-Kyung Cha, Sun-Young Im

**Affiliations:** ^1^Department of Psychology, Hallym University, Chuncheon-si, Republic of Korea; ^2^Department of Nursing Science, Sunmoon University, Asan-si, Republic of Korea

**Keywords:** COVID-19 pandemic, nurses, posttraumatic growth, burnout, PTSD, posttraumatic stress disorder

## Abstract

**Objective:**

The study aims to investigate factors that prevent burnout (BO) and symptoms of posttraumatic stress disorder (PTSD) while facilitating posttraumatic growth (PTG) among nurses combating the coronavirus disease 2019 (COVID-19) pandemic, with the purpose of validating the mediating effects of PTG.

**Methods:**

A total of 247 nurses who provided patient care during the COVID-19 pandemic were enrolled, and a questionnaire was used to measure BO, PTSD, and PTG, data on deliberate rumination, emotional expression, adaptive cognitive emotion regulation (CER), maladaptive CER, and social support. The mediation path models for the effects of the predictors on BO and PS through the mediation of PTG were analyzed using the R Lavaan package.

**Results:**

The results showed that deliberate rumination, emotional expression, and adaptive CER significantly increased PTG, while PTG significantly reduced BO and PTSD symptoms (PSs). However, maladaptive CER did not have a significant effect on PTG and only had significant direct effects on BO and PS. Bootstrapping confirmed that PTG significantly mediated the effects of all predictors. It partially mediated the effects of deliberate rumination and adaptive CER and completely mediated the effects of emotional expression.

**Conclusion:**

Based on the results, it has been supported that deliberate rumination, emotional expression, and adaptive CER should be addressed as important variables in psychological interventions addressing nurses’ adversities during the pandemic. These variables can prevent BO and PS by facilitating PTG.

## 1 Introduction

Since the outbreak of the coronavirus disease 2019 (COVID-19) in 2019, more than 30 million confirmed cases have been recorded in South Korea (1). Although several studies investigated the psychological repercussions of Severe Acute Respiratory Syndrome in 2002 and the Middle East Respiratory Syndrome in 2013, during the 3 years of COVID-19, there has been a marked gap in empirical research needed to prevent psychological burnout and protect the mental health of healthcare professionals working at the frontlines of the pandemic. It is important to note that PTSD and PTG can be found simultaneously in individuals (2–4). This indicates that these concepts are not entirely opposing phenomena and suggests that both may occur together during the pandemic. We employ a positive psychological approach, focusing not simply on minimizing the suffering and side effects but going beyond to overcoming the crisis and utilizing it as an opportunity for personal growth and development. *Posttraumatic growth* (PTG) refers to positive qualitative changes attained after a crisis beyond merely recovering to the pre-crisis level (5). It is not only a product of overcoming trauma but also serves as a protective factor against burnout, thus enhancing resilience (6).

In a study on tertiary hospital nurses who provided direct patient care during the pandemic, Lee et al. (7) reported that 63.9% of nurses had experienced PTSD, while 49.7% had clinical depression. A meta-analysis on the prevalence of PTSD during the COVID-19 pandemic reported that, in particular, nurses who provided direct patient care were experiencing high levels of stress and a high prevalence of PTSD (8). In addition, Martin et al. (9) reported that a high workload and unprecedented levels of burnout (BO) during the pandemic caused substantial levels of stress among the US nursing population, particularly among the younger and less experienced nurses, leading to an overall 3.3% decline in the I.S. nursing workforce over a 2-year period. The issues related to this work overload appear to be consistent not only among nurses directly handling infected patients but also among administrative staff (10). These results suggest that nurses in clinical practice would be affected by serious psychological BO and suffering. Healthcare professionals working at the frontlines of the pandemic faced higher levels of physical and emotional BO owing to more frequent exposures to pain from the loss of patients and colleagues and frequent infection risks (11), with the highest rate of BO reported among nurses (12).

A study on nurses’ PTG during the COVID-19 pandemic showed that nurses at the frontlines experienced moderate-to-severe psychological stress responses and that COVID-19-related stress leads to positive changes through deliberate rumination (13). In addition, studies have reported that PTG is negatively associated with psychological distress in frontline healthcare professionals (14). According to a study on the impact of PTG on psychological distress after COVID-19, the level of depression and anxiety declines with increasing PTG (15), and PTG is negatively associated with psychological distress in frontline healthcare professionals battling COVID-19 (13). Moreover, Hamama-Raz et al. (16) reported that PTG is negatively proportional to BO caused by job-related stress in nurses. PTG is a term that not only signifies the enhancement of individual psychological resources or abilities but also denotes the process of coping strategies for severe stress (17). PTG can serve as a coping strategy to shield individuals from the impact of a pandemic, allowing them to emerge from distress intact. We infer that PTG has a protective potential against deteriorations of psychological health and BO in healthcare professionals facing extreme psychological distress from COVID-19.

Cognitive emotion regulation (CER) refers to cognitive coping strategies, which enable an individual to control and not be overwhelmed by their emotions due to threatening or stressful events (18). Garnefski and colleagues delineated nine specific cognitive strategies, namely acceptance, positive reappraisal, positive refocusing, refocusing on planning, putting into perspective, self-blame, blaming others, rumination, and catastrophizing. Subsequent empirical research, including a comprehensive meta-analysis by Aldao et al. (19), has demonstrated that maladaptive CER strategies such as self-blame, blaming others, rumination, and catastrophizing are more strongly associated with psychopathology than their adaptive counterparts and that mood-related disorders such as depression and anxiety disorders are more strongly connected to CER than other (20). Conversely, adaptive CER strategies, especially positive reappraisal, positive refocusing, and refocusing on planning, have shown positive correlations with PTG in studies by Im and Kwon (21) and Lee and Yang (22). Reflective rumination, examined by Cui et al. (13) and Romeo et al. (23), positively influences PTG in nurses. Research involving Korean nurses has consistently validated that deliberate rumination positively influences PTG (24, 25). According to the posttraumatic growth model of Tedeschi and Calhoun (26), individuals typically undergo intrusive rumination immediately after a traumatic event but ultimately achieve PTG through a process involving deliberate rumination, emotional recognition and expression, and social support. This pathway has been validated by Morris and Shakespeare-Finch (27) and Taku et al. (28). Emotional expression following trauma, as indicated by Manne et al. (29) and Song and Lee (30), facilitates PTG.

Moreover, social support is sometimes described as the major environmental resource influencing PTG (31). Ogińska-Bulik et al. (32) reported a positive correlation between PTG and social support in healthcare professionals, including nurses, and Aliche et al. (33) reported in a study on young adult terrorism survivors that social support directly increases PTG. A meta-analysis (34) of 217 studies shows a consistent positive relationship between social support and PTG, significant in both longitudinal and cross-sectional studies. In addition, Žukauskienė et al. (35) reported that social support predicts a higher level of PTG in victims of domestic violence by a spouse. These findings suggest that social support has an impact on PTG.

Factors known to affect PTG, namely CER strategies, deliberate rumination, and emotional expression, not only facilitate positive changes following a traumatic experience but also alleviate negative symptoms such as anxiety, depression, and BO. BO is a response arising from stressful events characterized by emotional exhaustion, reduced personal accomplishment, and a negative or cynical attitude toward others (36). Previous research indicates that adaptive CER negatively impacts BO (37) and generalized anxiety (38). Therefore, adaptive CER appears to potentially reduce the negative effects resulting from stress events, such as BO. Reflective rumination can reduce symptoms of depression, anxiety (39), and the risk of stress-related symptoms and BO (40) by promoting the processing of information and meaning related to traumatic events. This aligns with previous findings suggesting that deliberate thinking about traumatic events can mitigate psychological distress by modifying pathological thought patterns (41, 42). Hence, we can infer that the positive benefits of deliberate rumination could apply to healthcare professionals who experienced the COVID-19 pandemic distress as well.

Expressing one’s emotions toward a traumatic experience reduces anger, tension, and depression in trauma survivors (43), while suppressing emotions increases anxiety, depression, and PTSD symptoms (PSs) (44, 45). These results have also been documented in a study on nurses’ BO. In a study of clinical nurses in Korea, Park (46) reported a negative correlation between emotional expression and BO. In other words, the odds for BO were lower among nurses who express their emotions more often.

Based on the results of the literature review, we hypothesized that PTG facilitators will reduce BO and PS in nurses through the mediation of PTG. We aim to develop and validate a path model based on this hypothesis. PTG serves as a buffer against BO and PS, and causal variables that promote PTG, such as deliberate rumination, emotional expressivity, and adaptive CER, are included in the model. In other words, PTG acts as a mediator in the model. Additionally, we have considered covariates that influence psychological variables. For example, religion has been associated with high PTG in the Korean sample (47). While specific hypotheses regarding the effect of other covariates have not been established, considering the characteristics of participants and the specificity of the situations they are in would help in understanding the findings.

## 2 Materials and methods

### 2.1 Participants

An online survey using Google Forms was conducted from September to December 2022 with nurses of secondary hospitals and local hospitals in the provinces of Gyeonggi, Gangwon, and Chungcheong in South Korea. All participants were explained the study’s purpose, after which they signed an informed consent form. A total of 251 nurses were enrolled. Of these nurses, data from four participants were excluded because of having a value of *p* < 0.001 on the Mahalanobis distance (*D*^2^) test (48) or choosing only one response option on four or more instruments; data from 247 participants were therefore included in the final analysis.

The participants’ demographic characteristics are summarized in Table 1. Of all participants, 233 were female (94.33%) and 14 were male (5.67%). The mean age was 32.67 years (SD = 8.35, excluding one participant owing to no response). Other demographic characteristics assessed were marital status, religion, education, clinical career, type of work facility, and job position.

**Table 1:** Participants’ demographic characteristics (*N* = 247).

Variable		*n*	Percentage (%)
Sex	Female	233	94.33
	Male	14	5.67
Age (years) (*M* = 32.67, *SD* = 8.35)	20s	112	45.34
	30s	87	35.22
	40s	32	12.96
	50s	14	5.67
	60s	1	0.40
	No response	1	0.40
Marital status	Single	164	66.40
	Married	82	33.20
	Divorced	1	0.40
Religion	No	149	60.32
	Yes	98	39.68
Religion-detailed	Christian	63	25.51
	Catholic	28	11.34
	Buddhist	7	2.83
Highest education	Three-year college	22	8.91
	4-year college	194	78.54
	Graduate school	31	12.55
Total clinical career (years) (*M* = 9.06, *SD* = 7.89)	< 1 year	2	0.81
	1–5 years	107	43.32
	6–10 years	67	27.13
	11–15 years	25	10.12
	16–20 years	16	6.48
	≥ 20 years	30	12.15
Current healthcare facility	Secondary care	116	46.96
	Tertiary care	131	53.04
Current work unit	Medical	71	28.74
	ICU	48	19.43
	Surgical	43	17.41
	Other	33	13.36
	ED	26	10.53
	OR	9	3.64
	Artificial kidney unit	7	2.83
	Outpatient	4	1.62
	Neonatal	3	1.21
	PACU	2	0.81
	Psychiatric	1	0.40
Job position	Staff nurse	191	77.33
	Charge nurse	29	11.74
	≥ Nurse manager	27	10.93

ICU, intensive care unit; ED, emergency department; OR, operation room; PACU, post-anesthesia care unit.

### 2.2 Instruments

#### 2.2.1 Posttraumatic growth inventory

The Posttraumatic Growth Inventory (PTGI) was developed by Tedeschi et al. (49) to measure PTG—referring to growth attained following a painful experience beyond the pre-trauma levels. The original 25-item scale covers five domains. In our study, the PTGI-X, a Korean-translated and adapted version by Im (47), was used. The PTGI-X contains 18 items that assess three factors (changes of self-perception and personal strength, spiritual-existential change, and interpersonal relationship). The participants rate each item using a 6-point scale from 0 (never experienced) to 5 (experienced frequently). Aggregated scores range from 0 to 90, in which higher scores indicate more substantial growth. The internal consistency (Cronbach’s α) was 0.95 for the entire inventory and 0.82–0.94 for each factor in our study.

#### 2.2.2 Burnout assessment tool

The Burnout Assessment Tool (BAT) is a self-report questionnaire developed by Schaufeli et al. (50) to measure BO and adapted and validated in Korean by Cho (51). The 22-item Korean version has one less item from the original tool, and each item is rated on a 5-point Likert scale (0 “never” to 4 “always”). Aggregated scores range from 0 to 88, in which higher scores indicate increased severity of burnout. Four factors are used: exhaustion, mental distance, impaired emotional control, and impaired cognitive control. The internal consistency (Cronbach’s α) was 0.93 for the entire inventory and 0.77–0.87 for each factor in our study.

#### 2.2.3 PTSD checklist for DSM-5

The PTSD Checklist for Diagnostic and Statistical Manual of Mental Disorders, Fifth Edition (DSM-5) (PCL-5) is developed by Blevins et al. (52) to measure levels of PTSD symptoms occurring after a trauma event. We used the Korean version validated by Lee et al. (53). This 20-item scale is rated on a 5-point Likert scale. Aggregated scores range from 0 to 80, in which higher scores indicate more severe symptoms. The cutoff score for the Korean version of the PCL-5 is 26, and participants who scored above this point were classified as the PTSD group (54). This cutoff score is relatively lower than those observed in clinical populations in other studies, which suggest cutoffs of 41 (55) and 37 (56). The PCL was originally developed based on the diagnostic criteria of DSM-5, considering the four factors: intrusion, avoidance, negative alterations in cognition and mood, and hyperarousal. Recent empirical advancements, however, advocate for a seven-factor model of PTSD—re-experiencing, avoidance, negative affect, anhedonia, externalizing behaviors, anxious arousal, and dysphoric arousal—as providing a superior fit. This model, supported by both theoretical and empirical evidence, represents a significant evolution in having the best fit (54, 57, 58). The internal consistency (Cronbach’s α) was 0.95 for the entire inventory in our study.

#### 2.2.4 Event-related rumination inventory

The Event-Related Rumination Inventory (ERRI) is a self-report scale developed by Cann et al. (59) to measure intrusive and deliberate rumination, which refers to repeated re-living of a shocking or painful event. There are 10 items for each factor, and each item is rated on a 4-point Likert scale. In the present study, we only used 10 items for deliberate rumination used in the Korean-validated version by Ahn et al. (60). Aggregated scores range from 0 to 30, in which higher scores indicate more deliberate rumination. The internal consistency (Cronbach’s *α*) was 0.94 for the deliberate rumination subscale.

#### 2.2.5 Emotional expressivity scale

The Emotional Expressivity Scale was developed by Kring et al. (61) to measure emotional expression, which refers to the degree to which one expresses positive and negative emotions. In the present study, we used the scale translated by Han (62) after modifying it for the COVID-19 situation. This single-factor scale contains eight items, and each item is rated on a 5-point Likert scale. Aggregated scores range from 0 to 32, in which higher scores indicate more emotional expression. The internal consistency (Cronbach’s α) was 0.64 in this study.

#### 2.2.6 Cognitive emotion regulation questionnaire

The Cognitive Emotion Regulation Questionnaire (CERQ) was developed by Garnefski et al. (18) to measure CER strategies for the regulation of emotions pertaining to an event. We used the Korean version validated by Ahn et al. (20). The tool contains 35 items, and each item is rated on a 6-point Likert scale. The scale includes two subscales: adaptive regulation (acceptance, putting into perspective, positive refocusing, refocus on planning, and positive reappraisal) and maladaptive regulation (self-blame, catastrophizing, other-blame, rumination, intrusive, and negative). Aggregated scores range from 0 to 70 for adaptive factors and from 0 to 55 for maladaptive factors. The internal consistency (Cronbach’s α) for the entire inventory was 0.95, and it ranged from 0.76 to 0.90 for sub-factors.

#### 2.2.7 Multidimensional scale of perceived social support

The Multidimensional Scale of Perceived Social Support was developed by Zimet et al. (63) to measure positive resources that can be obtained from social relationships. This tool measures social support received from family, friends, or significant others, and we used the Korean version developed by Yune and Oh (64). This 12-item tool is rated on a 7-point Likert scale. Aggregated scores range from 0 to 72, in which higher scores indicate more considerable social support. The internal consistency (Cronbach’s α) was 0.95 for the entire inventory and 0.94–0.96 for each factor in our study.

### 2.3 Analysis

First, descriptive statistics were reported, and skewness and kurtosis of the variables were examined to assess the multivariate normality assumption.

Second, an analysis of variance (ANOVA) and *t*-test were conducted to explore covariates potentially affecting variables in the mediation model. These included demographics and COVID-19-related job characteristics. Post-hoc analysis for the ANOVA was conducted using Scheffe’s method.

Third, we conducted model comparisons to select the causal variables to be included in the mediation model. This step was taken to determine if the variables (deliberate rumination, emotional expression, adaptive CER, maladaptive CER, and social support) theoretically believed to predict PTG, BO, and PS based on existing literature statistically significantly predict PTG, BO, and PS and to ensure parsimony of the mediation model. Model comparison was carried out using the Type III Sum of Squares (SS) method, which involved comparing the full model with restricted models where each independent variable was omitted one by one to identify variables causing significant residual changes in the model. This method is useful for exploring significant variables regardless of the order of entry of independent variables and simplifying the model. To diagnose multicollinearity in each model, the variance inflation factor (VIF) was calculated. In the PTG regression model, adjusted generalized VIF [GVIF^1/(2*df)^; (65)] was used due to the inclusion of a categorical independent variable of three levels (highest education). If the VIF exceeds 10 or the GVIF^1/(2*df)^ exceeds 2, it is interpreted as an indication of multicollinearity (65, 66).

Finally, we validated the mediation model using the covariates and causal variables selected in the first and second steps. Since covariates were all categorical variables, they were dummy-coded, and they were set to estimate the effects on causal, mediating, and outcome variables. We used the robust maximum likelihood method, and missing data were processed with full information maximum likelihood (FIML). One item containing 38 missing values was from the Event-Related Rumination Inventory.

## 3 Results

### 3.1 Descriptive statistics and correlation analysis

Table 2 shows the descriptive statistics of major variables and the correlations among the variables. The absolute values of skewness for all variables were less than 2, and the absolute values of kurtosis were less than 7, meeting the criteria for multivariate normality suggested by Curran et al. (67). According to the cutoff score of PCL-5 (54), 47.81% of the participants in this study were classified as the PTSD group. PTG was not significantly correlated with BO (*r* = −0.10, *p* = 0.100) and PS (*r* = 0.02, *p* = 0.774) but was significantly positively correlated with deliberate rumination (*r* = 0.57, *p* < 0.001), emotional expression (*r* = 0.32, *p* < 0.001), adaptive CER (*r* = 0.54, *p* < 0.001), maladaptive CER (*r* = 0.28, *p* < 0.001), and social support (*r* = 0.23, *p* < 0.001). There was a strong correlation between BO and PS (*r* = 0.69, *p* < 0.001), and both were significantly positively correlated with deliberate rumination (BO: *r* = 0.21, *p* < 0.001; PS: *r* = 0.39, *p* = 0.002) and maladaptive CER (BO: *r* = 0.43, *p* < 0.001; PS: *r* = 0.56, *p* < 0.001) and significantly negatively correlated with social support (BO: *r* = −0.19, *p* = 0.003; PS: *r* = −0.25, *p* < 0.001).

**Table 2:** Descriptive statistics and correlations.

Variables	1	2	3	4	5	6	7	8
Correlations								
1. PTG	1							
2. BO	−0.10	1						
3. PS	0.02	0.69^***^	1					
4. DR	0.57^***^	0.21^**^	0.39^***^	1				
5. EE	0.32^***^	−0.12	−0.11	0.10	1			
6. ACER	0.54^***^	0.01	0.08	0.64^***^	0.25^***^	1		
7. MCER	0.28^***^	0.43^***^	0.56^***^	0.50^***^	0.05	0.43^***^	1	
8. SS	0.23^***^	−0.19^**^	−0.25^***^	0.07	0.34^***^	0.26^***^	−0.15^*^	1
Descriptive statistics (total score)								
Missing	0	0	0	38	0	0	0	0
*M*	42.19	41.59	25.23	13.52	14.39	39.35	18.97	54.80
SD	19.21	14.39	17.22	7.45	4.80	15.67	13.07	14.90
Min.	0	5	0	0	0	0	0	2
Max.	83	80	72	30	27	70	55	72
Skewness	−0.21	0.21	0.41	0.11	−0.31	−0.50	0.53	−1.08
Kurtosis	−0.66	−0.19	−0.49	−0.59	0.40	−0.34	−0.34	1.03

^*^p < 0.05, ^**^p < 0.01, ^***^p < 0.001. PTG, posttraumatic growth; BO, burnout; PS, PTSD symptoms; DR, deliberate rumination; EE, emotional expression; ACER, adaptive CER; MCER, maladaptive CER; SS, social support.

### 3.2 Comparison of major variables according to demographic characteristics

According to demographic characteristics, the differences in the variables were analyzed using a *t*-test or ANOVA (Table 3). The results indicated that age, education level, religion, type of healthcare facility, current job position, prior work experience in a screening center, prior work experience in a residential treatment center, shift work during the COVID-19 pandemic, and prior completion of COVID-19-related infection control education had a significant effect on one or more study variables. More specifically, education level influenced PTG, *F*(2, 244) = 3.053, *p* = 0.049, *η*^2^ = 0.024, and religion influenced PTG, *t*(245) = −3.912, *p* < 0.001, *d* = 0.510, 95% C.I. [0.250, 0.768], BO, *t*(245) = 2.286, *p* = 0.023, *d* = 0.298, 95% C.I. [0.041, 0.554], and deliberate rumination, *t*(207) = −2.360, *p* = 0.019, *d* = 0.338, 95% C.I. [0.055, 0.621]; Type of healthcare facility influenced PTG, *t*(245) = 2.248, *p* = 0.025, *d* = 0.287, 95% C.I. [0.035, 0.538], and current job position influenced PTG, *t*(245) = −2.885, *p* = 0.004, *d* = 0.438, 95% C.I. [0.138, 0.738], BO, *t*(245) = 2.674, *p* = 0.008, *d* = 0.406, 95% C.I. [0.106, 0.706], PS, *t*(110.64) = 2.373, *p* = 0.019, *d* = 0.338, 95% C.I. [0.019, 0.618], and maladaptive CER, *t*(130.50) = 3.199, *p* = 0.002, *d* = 0.435, 95% C.I. [0.097, 0.697]. An imbalance in sample size across groups was found for certain variables, such as gender, making it difficult to test the effects of these variables.

**Table 3:** Comparison of major measurement variables according to demographic characteristics (one-way ANOVA).

Dependent variable	Independent variable	Level (group)	M (SD)	*F* or *t* (*df*)	*η^2^* or *d*	*Post hoc*_Scheffe_
PTG	Education^F^	3-year college^a^	39.59 (20.19)	3.053^*^ (2, 244)	0.024	*n.s.*
		4-year college^b^	41.24 (18.24)			
		Graduate school^c^	50.00 (22.93)			
	Religion^S^	No^a^	38.45 (18.08)	−3.912^***^ (245)	0.510	b > a
		Yes^b^	47.97 (19.55)			
	Type of healthcare facility^S^	Secondary^a^	45.09 (17.51)	2.248^*^ (245)	0.287	a > b
		Tertiary^b^	39.63 (20.31)			
	Job position^S^	Staff nurse^a^	40.31 (18.80)	−2.885^**^ (245)	0.438	b > a
		Charge nurse or higher^b^	48.61 (19.37)			
Burnout	Religion^S^	No^a^	43.26 (15.00)	2.286^*^ (245)	0.298	a > b
		Yes^b^	39.01 (13.05)			
	Job position^S^	Staff nurses^a^	42.90 (14.12)	2.674^**^ (245)	0.406	a > b
		Charge nurse or higher^b^	37.13 (14.51)			
PTSD symptoms	Job position^W^	Staff nurse^a^	26.47 (17.85)	2.373^*^ (110.64)	0.338	a > b
		Charge nurse or higher^b b^	21.02 (14.23)			
Deliberate rumination	Religion^S^	No^a^	12.60 (7.07)	−2.360^*^ (207)	0.338	b > a
		Yes^b^	15.09 (7.85)			
Maladaptive CER	Job position^W^	Staff nurse^a^	20.14 (13.76)	3.199^**^ (130.50)	0.435	a > b
		Charge nurse or higher^b^	15.00 (9.43)			

^*^p < 0.05, ^**^p < 0.01, ^***^p < 0.001; d = Cohen’s d; Based on Levene equal variance test results, equal variance was assumed or not assumed: ^F^Fisher’s test (ANOVA for equal variance), ^S^Student’s test (*t*-test for equal variance), ^W^Welch’s test (ANOVA or *t*-test for unequal variance).

### 3.3 Predictors of PTG, BO, and PS

Table 4 shows the results of regression analysis to investigate the significant predictors of PTG, BO, and PS. PTG was statistically significantly influenced by religion, *F*(1, 198) = 6.153, *p* = 0.014, current job position, *F*(1, 198) = 5.880, *p* = 0.016, deliberate rumination, *F*(1, 198) = 26.772, *p* < 0.001, emotional expression, *F*(1, 198) = 15.673, *p* < 0.001, and adaptive CER, *F*(1, 198) = 6.620, *p* = 0.011. In the regression model for PTG, the GVIF^1/(2* *df*)^ values ranged from 1.06 for the type of work facility to 1.41 for adaptive CER, indicating no multicollinearity issues. BO was statistically significantly influenced by PTG, *F*(1, 200) = 4.978, *p* = 0.027, deliberate rumination, *F*(1, 200) = 8.638, *p* = 0.004, adaptive CER, *F*(1, 200) = 7.297, *p* = 0.007, and maladaptive CER, *F*(1, 200) = 44.942, *p* < 0.001. PS was statistically significantly influenced by PTG, *F*(1, 201) = 3.966, *p* = 0.048, deliberate rumination, *F*(1, 201) = 25.691, *p* < 0.001, adaptive CER, *F*(1, 201) = 14.826, *p* < 0.001, and maladaptive CER, *F*(1, 201) = 67.298, *p* < 0.001. In the models for BO (VIF ranged from 1.10 for religion to 2.22 for deliberate rumination) and PS (VIF ranged from 1.12 for job position to 2.21 for deliberate rumination), there were no issues of multicollinearity.

**Table 4:** Model comparison for PTG, burnout, and PTSD symptoms (type III method).

Restricted variables	*Δ*SS	*Δdf*	*F*	*p*
Dependent variable: PTG				
Highest education (Ref = 3-year college)	103.65	2	0.255	0.775
Religion (Ref = No)	1250.35	1	6.153^*^	0.014
Type of healthcare facility (Ref = secondary care facility)	352.84	1	1.736	0.189
Job position (Ref = Staff nurse)	1194.87	1	5.880^*^	0.016
Deliberate rumination	5440.56	1	26.772^***^	< 0.001
Emotional expression	3185.14	1	15.673^***^	< 0.001
Adaptive CER	1345.27	1	6.620^*^	0.011
Maladaptive CER	88.01	1	0.433	0.511
Social support	589.01	1	2.898	0.090
Error variance	40237.62	198		
Dependent variable: burnout				
Religion (Ref = No)	496.12	1	3.339	0.069
Job position (Ref = Staff nurse)	126.42	1	0.851	0.357
PTG	739.56	1	4.978^*^	0.027
Deliberate rumination	1283.37	1	8.638^**^	0.004
Emotional expression	162.76	1	1.096	0.297
Adaptive CER	1084.12	1	7.297^**^	0.007
Maladaptive CER	6676.74	1	44.942^***^	< 0.001
Social support	145.05	1	0.976	0.324
Error variance	29712.82	200		
Dependent variable: PTSD symptoms				
Job position (Ref = Staff nurse)	1.00	1	0.006	0.940
PTG	697.94	1	3.966^*^	0.048
Deliberate rumination	4520.88	1	25.691^***^	< 0.001
Emotional expression	110.93	1	0.630	0.428
Adaptive CER	2609.02	1	14.826^***^	< 0.001
Maladaptive CER	11842.69	1	67.298^***^	< 0.001
Social support	124.36	1	0.707	0.402
Error variance	35370.69	201		

^*^p < 0.05, ^**^p < 0.01, ^***^p < 0.001. Analysis of deliberate rumination and maladaptive CER are equal to that in Table 5 and thus were omitted.

### 3.4 Validation of mediation model

**Figure 1:**
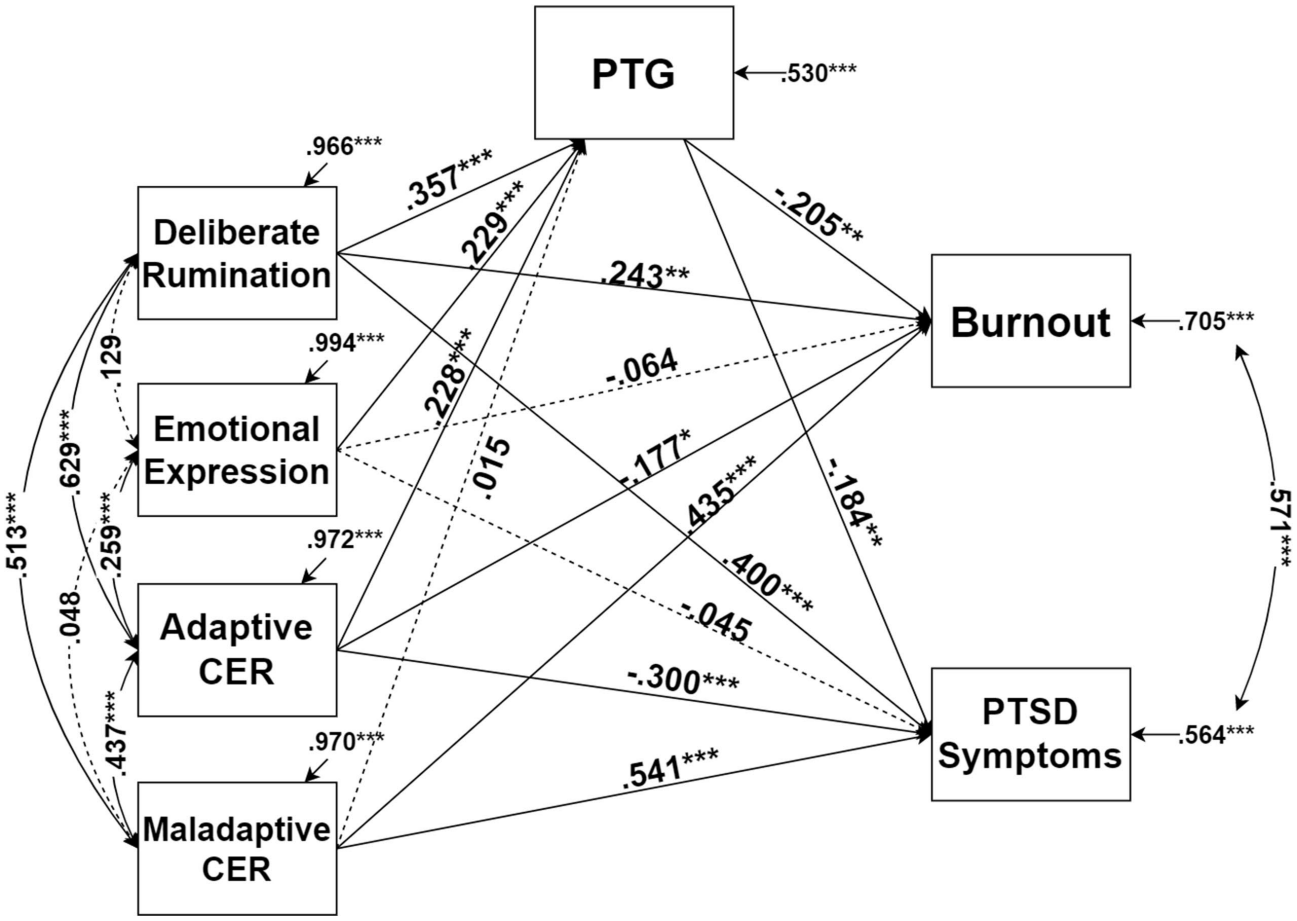
Result of path modeling. ^*^p < 0.05, ^**^p < 0.01, ^***^p < 0.001. All coefficients in the figure are standardized, and dotted lines are statistically insignificant coefficients.

Finally, we conducted a path model analysis to determine whether the predictors affect BO and PS through PTG with the inclusion of demographic variables in the model. The results of the mediation model analysis are shown in Figure 1 and Tables 5, 6. Bootstrapping (Table 6) was performed to statistically analyze the mediating effects. Deliberate rumination, emotional expression, and adaptive CER significantly predicted BO and PS with the inclusion of demographic variables in the model. The total effect of all causal variables on BO was significant (*p* < 0.05), and the total effect of all causal variables excluding emotional expression on PS was significant (*p* < 0.001). In summary, the effect of deliberate rumination and adaptive CER on BO and PS was partially mediated by PTG, and the effect of emotional expression was completely mediated by PTG.

**Table 5:** Effects of covariates within the path model.

Outcome variable	Covariate		Regression coefficient		95% CI^a^		
	Variable	Level	Ust.	C.R.	Std.	L.L.	U.L.
Deliberate rumination	Religion^b^	Yes	2.105	2.109^*^	0.286	0.076	4.213
	Job position^c^	Charge nurse or higher	−0.724	−0.567	−0.098	−3.085	1.620
	Highest education^d^	4-year college	−0.679	−0.393	−0.092	−4.866	3.418
		Graduate school	2.016	0.914	0.274	−2.876	7.050
Emotional expression	Religion^b^	Yes	−0.414	−0.659	−0.086	−1.589	0.771
	Job position^c^	Charge nurse or higher	0.040	0.049	0.008	−1.402	1.449
	Highest education^d^	4-year college	0.687	0.639	0.143	−1.095	2.435
		Graduate school	−0.001	−0.001	0.000	−2.547	2.670
Adaptive CER	Religion^b^	Yes	2.531	1.469	0.190	−0.833	5.990
	Job position^c^	Charge nurse or higher	−1.525	−0.688	−0.115	−5.840	2.655
	Highest education^d^	4-year college	3.244	1.100	0.244	−4.071	10.230
		Graduate school	7.854	2.042^*^	0.591	−0.859	16.147
Maladaptive CER	Religion^b^	Yes	1.340	0.794	0.103	−2.054	4.681
	Job position^c^	Charge nurse or higher	−5.473	−2.518^*^	−0.420	−9.091	−2.044
	Highest education^d^	4-year college	−0.933	−0.323	−0.072	−7.636	5.570
		Graduate school	−0.345	−0.092	−0.026	−8.219	7.657
PTG	Religion^b^	Yes	6.233	3.310^**^	0.325	2.376	10.085
	Job position^c^	Charge nurse or higher	7.145	2.953^**^	0.373	2.467	11.761
	Highest education^d^	4-year college	0.700	0.218	0.037	−6.725	8.523
		Graduate school	1.254	0.300	0.065	−7.732	10.299
Burnout	Religion^b^	Yes	−3.581	−2.174^*^	−0.249	−6.882	−0.274
	Job position^c^	Charge nurse or higher	−1.173	−0.555	−0.082	−5.833	3.411
	Highest education^d^	4-year college	−3.058	−1.113	−0.213	−9.962	3.244
		Graduate school	−4.659	−1.298	−0.324	−12.905	3.202
PTSD symptoms	Religion^b^	Yes	0.548	0.308	0.032	−3.167	4.050
	Job position^c^	Charge nurse or higher	−0.732	−0.321	−0.043	−5.152	3.541
	Highest education^d^	4-year college	−0.359	−0.121	−0.021	−8.139	7.055
		Graduate school	−0.420	−0.109	−0.024	−8.971	8.426

^*^p < 0.05, ^**^p < 0.01, ^***^p < 0.001. ^a^Bootstrapping (*N* = 5,000). ^b^Reference: no religion. ^c^Reference: staff nurse. ^d^Reference: 3-year college. Ust., unstandardized coefficient; Std., standardized coefficient for all variables other than independent variables (Std.nox), C.R., critical ratio.

**Table 6:** Effect decomposition (total effect, mediating effect, and direct effect) for the path model and bootstrapping results.

Effect on DVs	Causal variables								Mediating variable	
	Deliberate rumination		Emotional expression		Adaptive cognitive emotion regulation		Maladaptive cognitive emotion regulation		Posttraumatic growth (PTG)	
	Coefficients	95% C.I.^a^	Coefficients	95% C.I.^a^	Coefficients	95% C.I.^a^	Coefficients	95% C.I.^a^	Coefficients	95% C.I.^a^
	Ust.(Std.)	L.L. ~ U.L.	Ust.(Std.)	L.L. ~ U.L.	Ust.(Std.)	L.L. ~ U.L.	Ust.(Std.)	L.L. ~ U.L.	Ust.(Std.)	L.L. ~ U.L.
Posttraumatic growth (PTG)—mediating variable (*R*^2^ = 0.470)										
DE	0.930(0.357)^***^	0.553 ~ 1.308	0.914(0.229)^***^	0.469 ~ 1.334	0.328(0.228)^***^	0.130 ~ 0.533	0.022(0.015)	−0.152 ~ 0.191	—	—
ME	—	—	—	—	—	—	—	—	—	—
TE	0.930(0.357)^***^	0.553–1.308	0.914(0.229)^***^	0.469 ~ 1.334	0.328(0.228)^***^	0.130 ~ 0.533	0.022(0.015)	−0.152 ~ 0.191	—	—
Burnout (BO)—outcome variable (*R*^2^ = 0.295)										
DE	0.473(0.243)^**^	0.146–0.813	−0.190(−0.064)	−0.531 ~ 0.143	−0.191(−0.177)^*^	−0.391 ~ −0.001	0.479(0.435)^***^	0.345 ~ 0.607	−0.153(−0.205)^**^	−0.256 ~ −0.048
ME	−0.143(−0.073)^*^	−0.275 ~ −0.040	−0.140(−0.047)^*^	−0.268 ~ −0.039	−0.050(−0.047)^*^	−0.104 ~ −0.012	−0.003(−0.003)	−0.032 ~ 0.025	—	—
TE	0.330(0.170)^*^	0.009 ~ 0.667	−0.331(−0.110)^*^	−0.657 ~ −0.002	−0.242(−0.224)^**^	−0.433 ~ −0.052	0.476(0.432)^***^	0.340 ~ 0.605	−0.153(−0.205)^**^	−0.256 ~ −0.048
PTSD symptom (PS)—outcome variable (*R*^2^ = 0.436)										
DE	0.933(0.400)^***^	0.545 ~ 1.322	−0.162(−0.045)	−0.556 ~ 0.233	−0.388(−0.300)^***^	−0.583 ~ −0.205	0.713(0.541)^***^	0.559 ~ 0.865	−0.165(−0.184)^**^	−0.290 ~ −0.043
ME	−0.153(−0.066)^*^	−0.312 ~ −0.036	−0.150(−0.042) ^*^	−0.305 ~ −0.032	−0.054(−0.042)^*^	−0.116 ~ −0.010	−0.004(−0.003)	−0.034 ~ 0.029	—	—
TE	0.780(0.334)^*^	0.387 ~ 1.154	−0.313(−0.087)	−0.685 ~ 0.054	−0.442(−0.342)^***^	−0.627 ~ −0.260	0.710(0.539)^***^	0.554 ~ 0.865	−0.165(−0.184)^**^	−0.290 ~ −0.043

^**^p < 0.01, ^***^p < 0.001. DVs, dependent variables; TE, total effect; ME, mediating effect; DE, direct effect. ^a^Bootstrapping (*N* = 5,000). Ust., unstandardized coefficient; Std., standardized coefficient (Std. all in Lavaan package).

## 4 Discussion

This study explored the variables that may influence PTG and BO in nurses during the COVID-19 pandemic and examined whether variables such as deliberate rumination, emotional expression, and adaptive CER can reduce BO and PS through the mediation of PTG. The major findings are summarized and discussed below.

First, we identified the predictors of BO, PS, and PTG. Initially, deliberate rumination, emotional expression, and adaptive CER were significant predictors of PTG. The roles of deliberate rumination (13, 23–25), emotional expression (27, 29, 30), and adaptive CER (22) as predictors of PTG have already been supported by previous studies. The findings of our study emphasize that these predictors are also essential factors in facilitating PTG in nurses. Next, deliberate rumination, adaptive CER, maladaptive CER, and PTG had significant direct effects on BO and PS. Of them, adaptive CER directly reduced BO and PS. Our result that adaptive CER alleviates job-related BO and relevant stress symptoms is consistent with previous findings (37). In other words, these are in line with the interpretation of Ehlers and Steil (41) that adaptive CER and deliberate thoughts about a trauma (or extreme stress) can modify pathological thinking patterns and thus reduce psychological distress. However, maladaptive CER increased BO and PS. Maladaptive CER related to maladaptive coping, such as avoidance and denial, prevents individuals from confronting negative experiences, thus hindering problem resolution in challenging situations. As a result, they may contribute to maintaining or even increasing psychological distress. Contrary to the hypothesis of this study, however, social support was not a significant causal variable. While many studies support the significant effect of social support, the role of social support is not simple. Recently, Saegert and Carpiano (68) revealed that social support does not solely bring positive effects but rather complex outcomes in conjunction with other factors, and some supportive relationships may have negative effects. For example, conflicts may arise when supporters become exhausted from providing sustained support over time (69). Social support arises within relationships with others, and the dynamics occurring within those relationships shape social support (70). In this regard, the social support reported by the participants may not have aligned well with the specific contextual characteristics of the pandemic. Additionally, one of the factors of social support measured in this study is the support of family, but it has been pointed out that family members may not be a helpful resource for all stressors due to the significant obligations and potential for conflict among them (70).

As opposed to our hypothesis, deliberate rumination had a direct effect on BO and PS. It is important to note that these findings are based on data collected in the unique context of the COVID-19 pandemic. During the ongoing pandemic, the healthcare system faced numerous challenges, and nurses had to deal with many difficult and stressful experiences. Therefore, it is possible that during the pandemic, engaging in more reflection and rumination about their own challenging experiences may actually have consumed their psychological energy, ultimately leading to increased BO and PS. For example, deliberately focusing on distressing memories and thoughts instead of avoiding them induces pain and stress. While deliberate rumination may lead to experiencing PTG in the long term, in the short term, deliberate rumination may not have been as effective in addressing nurses’ burnout and PTSD symptoms related to the pandemic as expected. Previous studies have already discovered that factors that had been protective prior to the COVID-19 pandemic (e.g., coping flexibility) had adverse effects during the pandemic (71). In other words, it is essential to consider the context of an individual’s situation to understand the roles of a particular variable (72). It is important to note that although deliberate rumination directly increases BO and PS, it also lowers BO and PS if PTG is increased. Given that deliberate rumination is associated with increasing PTG, it seems important to utilize deliberate rumination for patients experiencing PTSD or burnout. However, since deliberate rumination can induce pain, it appears necessary to employ it with psychological resources and abilities to endure that pain. For example, in psychotherapy, patients may find confronting psychological problems distressing, yet confronting them can be highly beneficial in resolving the problem [e.g., (73)].

Second, the effects of adaptive CER and deliberate rumination on BO and PS were partially mediated by PTG. Deliberate rumination involves reflecting on their painful experiences and contemplating the meaning behind those experiences. This reflection may encourage people to find positive opportunities for growth amid the psychological distress caused by negative events, ultimately leading to a reduction in BO and PS. Adaptive coping is crucial in alleviating the distress associated with negative experiences and achieving PTG. In contrast to maladaptive CER, such as avoidance, catastrophizing, and self-blame, which hinder cognitive processing of negative experiences and increase BO and PS, adaptive CER encourages individuals to reinterpret events positively, accept them, and make efforts to solve problems. These effects promote experiencing PTG in the aftermath of negative events and naturally reduce BO and PS (41).

In contrast to other causal variables, the effects of emotional expression on BO and PS were completely mediated by PTG. Emotional expression relieves accumulated emotional burdens and facilitates deliberate rumination through actions such as purification (26). As a result, it is anticipated to increase PTG and reduce BO and PS. However, emotional expression alone may not be adequate in reducing negative outcomes (BO and PS). Emotional expression is a kind of self-disclosure, and self-disclosure is one of the precursors to PTG (74). If elements such as cognitive changes follow emotional expression, they can lead to positive subsequent changes (75). In other words, it can reduce BO and PS if it leads to PTG through cognitive processes such as reflection, but if PTG is not achieved, emotional expression itself (e.g., expressing pain and distress) appears to be inadequate in directly reducing BO and PS.

The finding that deliberate rumination, emotional expression, and adaptive CER facilitate PTG, which in turn leads to reduced BO and PS, carries significant clinical implications. While various psychological treatment programs have been developed and utilized to address trauma and severe stress, most of them have primarily focused on reducing maladaptive symptoms. However, there is a growing trend toward programs that not only aim to reduce maladaptive symptoms but also emphasize personal growth and maturity in response to adversity [e.g., (76, 77)]. Nevertheless, the development and utilization of psychological therapy programs to address the challenges experienced by healthcare workers during the COVID-19 pandemic are not yet widespread. While programs for the general population are being developed and implemented extensively, there is a shortage of programs addressing the unique experiences of healthcare professionals. The results of this study, in conjunction with previous research, can serve as a basis for the development and utilization of programs aimed at managing the BO and psychological distress of frontline healthcare workers, including nurses, and facilitating their growth. By leveraging factors such as deliberate rumination, emotional expression, and adaptive CER, it is possible to prevent maladaptive symptoms and promote positive psychological growth and change. For instance, nurses can be encouraged to transform their habitual maladaptive rumination on the job into adaptive and reflective forms (78) and subsequently engage in problem-solving coping strategies. Some studies also propose methods for alleviating maladaptive rumination by facilitating the mental construction of novel scenarios in the immediate and distant future (79). Furthermore, providing nurses with opportunities to appropriately express suppressed emotions such as anger, frustration, and worry during their duties [e.g., (80)] could increase their objective self-understanding and objective perspective on issues, thereby alleviating BO and the associated psychological distress (81).

Religion emerged as the second most influential demographic characteristic in our study. Participants who identified with a religion reported higher levels of DR and PTG and lower levels of BO compared to their non-religious counterparts. The role of religion in enhancing DR and PTG is well-supported by previous studies. For instance, religion may facilitate the PTG process by enabling individuals to find meaning in life, identify effective coping strategies and resources, and develop a revised personal narrative (82, 83). Additionally, positive religious coping, such as seeking spiritual support or maintaining a connection with God, predicted PTG following a disaster (84). Some religious doctrines view suffering not merely as a negative outcome but as an essential element for achieving purification, holiness, and preparation for the afterlife (85). Furthermore, various religions promote vocational callings or ethics (e.g., Luther’s doctrine of vocation). From this perspective, it is possible that religion facilitated individuals who suffered from a trauma or extremely stressful event to endure their suffering and deliberately ruminate the experience, thereby mitigating the impact of BO.

This study has a few limitations. First, the sample size was relatively small, given the complexity of the model being evaluated. Although most of the statistical analysis results yielded the expected or significant findings, there were instances in the model verification where some regression coefficients were statistically insignificant despite being reasonably large. Second, additional analysis was difficult for some demographic variables because of sample imbalances (e.g., female participants significantly outnumbered male participants at 233 to 14). Moreover, the marked differences in the number of participants across education levels (3-year college, 4-year college, and graduate school ranging from 22 to 194 participants) violated the strict assumption that group sizes should be equal in ANOVA. Next, the internal consistency of the Emotional Expressivity Scale was somewhat low. This could limit the interpretation of the findings of this study. It is anticipated that this may be due to the scale being developed too long ago and, therefore, not adequately measuring the construct of interest. In addition, it is necessary to include exposure to COVID-19-related trauma or infection patients as control variables, although several covariates selected through preliminary analysis were included in the model. By comparing the participants in this study with other nurses who were not exposed to trauma related to the COVID-19 pandemic or to the duties of treating infected patients, we can more accurately determine whether COVID-19 has impacted BO, PTSD, and PTG. Further research on this issue is needed. Finally, there are limitations related to the generalizability of the study findings. The cross-sectional design of this study limits the capacity to draw causal conclusions. Implementing a longitudinal design would help establish causal relationships between variables, which is essential for understanding the development of burnout, PTSD, and PTG over time. Additionally, our research is geographically confined to nurses working in South Korea. This limitation curtails the generalizability of our findings to other cultural or national contexts. A more inclusive study design involving participants from multiple countries, such as the approach taken by Chirico and Nucera (86), would potentially offer a broader perspective and enhance the applicability of the results across different settings.

Despite these limitations, however, this study has the following implications. First, it highlights that while COVID-19 can be perceived negatively, it can also serve as a catalyst for positive change. By identifying the factors that promote PTG, this study offers direction on how to intervene with healthcare professionals during the COVID-19 pandemic. Second, it emphasizes the need for psychological interventions for healthcare professionals who are dealing with the challenges of the COVID-19 pandemic. Third, a comprehensive investigation was conducted on various factors that influence not only PTG but also BO and PTSD. This study covers a broad range of factors influencing PTG, BO, and PTSD. Finally, showing that PTG can serve as a buffer against BO and PTSD, this study provides evidence that a positive psychology perspective can be useful in psychological interventions for BO and PTSD. We hope that the results of this study will be valuable in providing psychological support to healthcare workers struggling with the demands of the pandemic and, ultimately, those who have experienced trauma or extreme stress events.

## 5 Conclusion

This study explored the variables that may influence posttraumatic stress disorder and burnout in healthcare professionals, such as nurses, during the COVID-19 pandemic. It highlights that it has examined the pathways through which these variables lead to reduced burnout through the mediation of posttraumatic growth. By fostering the factors that promote PTG, which serves as a buffer for BO and PS in healthcare professionals, they can achieve the benefits of psychological growth and improved mental health simultaneously. It is expected that the results of this study will be valuable in providing psychological support to healthcare workers struggling with the demands of the pandemic and, ultimately, those who have undergone traumatic or highly stressful events.

## Data availability statement

The raw data supporting the conclusions of this article will be made available by the authors, without undue reservation.

## Ethics statement

The studies involving humans were approved by the Hallym Institutional Review Board. The studies were conducted in accordance with the local legislation and institutional requirements. The participants provided their written informed consent to participate in this study.

## Author contributions

J-CS: Writing – original draft, Software, Project administration, Methodology, Investigation, Formal analysis, Data curation. S-KC: Writing – review & editing, Supervision, Data curation. S-YI: Writing – review & editing, Writing – original draft, Supervision, Project administration, Funding acquisition, Data curation, Conceptualization.
